# *Trichomonas* Transmembrane Cyclases Result from Massive Gene Duplication and Concomitant Development of Pseudogenes

**DOI:** 10.1371/journal.pntd.0000782

**Published:** 2010-08-03

**Authors:** Jike Cui, Suchismita Das, Temple F. Smith, John Samuelson

**Affiliations:** 1 Department of Molecular and Cell Biology, Boston University Goldman School of Dental Medicine, Boston, Massachusetts, United States of America; 2 Graduate Program in Bioinformatics, Boston University, Boston, Massachusetts, United States of America; 3 Department of Biomedical Engineering, Boston University, Boston, Massachusetts, United States of America; University of Pittsburgh, United States of America

## Abstract

**Background:**

*Trichomonas vaginalis* has an unusually large genome (∼160 Mb) encoding ∼60,000 proteins. With the goal of beginning to understand why some *Trichomonas* genes are present in so many copies, we characterized here a family of ∼123 *Trichomonas* genes that encode transmembrane adenylyl cyclases (TMACs).

**Methodology/Principal Findings:**

The large family of TMACs genes is the result of recent duplications of a small set of ancestral genes that appear to be unique to trichomonads. Duplicated TMAC genes are not closely associated with repetitive elements, and duplications of flanking sequences are rare. However, there is evidence for TMAC gene replacements by homologous recombination. A high percentage of TMAC genes (∼46%) are pseudogenes, as they contain stop codons and/or frame shifts, or the genes are truncated. Numerous stop codons present in the genome project G3 strain are not present in orthologous genes of two other *Trichomonas* strains (S1 and B7RC2). Each TMAC is composed of a series of N-terminal transmembrane helices and a single C-terminal cyclase domain that has adenylyl cyclase activity. Multiple TMAC genes are transcribed by *Trichomonas* cloned by limiting dilution.

**Conclusions/Significance:**

We conclude that one reason for the unusually large genome of *Trichomonas* is the presence of unstable families of genes such as those encoding TMACs that are undergoing massive gene duplication and concomitant development of pseudogenes.

## Author Summary

*Trichomonas vaginalis* is the only medically important protist (single-cell eukaryote) that is sexually transmitted. The ∼160-Mb *Trichomonas* genome contains more predicted protein-encoding genes (∼60,000) than the human genome. To begin to understand why there are so many copies of some genes, we chose here to study a large family of genes encoding unique transmembrane cyclases. Our most important results include the following. More than 100 transmembrane cyclase genes do not result from chromosomal duplications, because for the most part only the coding regions of the genes, rather than flanking sequences, are duplicated. Almost half of the transmembrane cyclase genes are pseudogenes, and these pseudogenes are polymorphic among laboratory strains of *Trichomonas*. Messenger RNAs for numerous transmembrane cyclases are expressed simultaneously, and representative cyclase domains have adenylyl cyclase activity. In summary, the large family of *Trichomonas* genes encoding transmembrane adenylyl cyclases results from massive gene duplication and concomitant development of pseudogenes.

## Introduction

*Trichomonas vaginalis*, the most important sexually transmitted protist, causes vaginitis in women and urethritis in men [1]–[3]. In addition, *Trichomonas* increases the risk of HIV transmission, pelvic inflammatory disease, and spontaneous abortion [4]. *Trichomonas* lives under microaerophilic conditions in the lumen of the vagina by means of fermentation enzymes that are present in a modified mitochondrion called the hydrogenosome [5]. This organelle lacks enzymes of oxidative phosphorylation but makes hydrogen, and many of its fermentation enzymes were acquired from bacteria by horizontal gene transfer [6]. *Trichomonas* causes vaginitis when the protist adheres to the host epithelium and changes from a flagellated to an ameboid form [7].

Recent whole genome sequencing showed an ∼160-Mb *Trichomonas* genome encoding ∼60,000 proteins [8]. This genome is bigger than those of many other medically important protists but is characteristic of trichomonads. One reason for the large *Trichomonas* genome is the presence of hundreds of DNA transposons that include mariner elements and Mavericks [9], [10]. Mavericks are of particular interest, because they are abundant, are ∼22-kb long, and so compose ∼30% of the genome. In addition, each Maverick contains 9 to 11 ORFs, such that Maverick proteins compose more than 50% of the predicted proteins of *Trichomonas*. Introns are rare and short, so the presence of large non-coding regions in *Trichomonas* genes cannot be an explanation for the large genome size [11].

We were interested in why some *Trichomonas* genes are present in so many copies and focused on one a large family of predicted transmembrane adenylyl cyclases (TMACs). These TMACs are of particular note because (1) they have a predicted topology different from those of other metazoan and protist transmembrane cyclases, and they appear to have originated via gene duplication in *Trichomonas* and closely related species (e.g. *Tritrichomonas* and *Paratrichomonas*; see below) [12]–[15], and (2) we discovered numerous in-frame stop codons and frame shifts in these genes, which made them a valuable dataset for exploring pseudogene evolution [16]–[20]. In addition to characterizing TMAC gene duplication and pseudogenes, we measured the mRNA levels of the TMAC genes and pseudogenes in trophozoites, and we determined whether recombinant cyclase domains from representative TMACs have adenylyl cyclase or guanylyl cyclase activity.

## Materials and Methods

### Bioinformatic identification of *Trichomonas* genes encoding putative transmembrane cyclases

The genome of *Trichomonas vaginalis* strain G3 has been sequenced to ∼6× redundancy, so that it is likely that the majority of genes have been predicted [8]. The predicted proteins of *Trichomonas* present at the NCBI or at TrichDB [21] were searched using BLASTP and cyclase domains from TMACs of *Dictyostelium discoideum*, *Homo sapiens*, and *Trypanosoma brucei*, as well as those of the TMGCs of *Homo sapiens* [12]–[14], [22]. We also used a full-length *Trichomonas* TMAC protein sequence (TVAG_350120) and BLASTP to search the predicted proteins of *Trichomonas* or used this TMAC and TBLASTN to search *Trichomonas* scaffolds in the database at J. Craig Venter Institute (JCVI) or the WGS database at the NCBI. Intact TMAC genes, apparent TMAC pseudogenes (see below), and partially sequenced TMAC genes due to assembly problems are listed in Data S1. The full length TMAC protein sequence and TBLASN was also used to search EST sequences at the NCBI from *Tritrichomonas foetus* and *Pentatrichomonas hominis*.

Transmembrane helices (TMHs) of TMACs were predicted using the Phobius combined transmembrane topology and signal peptide predictor [23]. Predicted proteins were examined for conserved domains using the CD search at the NCBI [24]. A representative set of 70 TMACs was aligned, and the conservation of sequences across the entire alignment was plotted using WebLogo [25]. Cyclase domains were aligned using MUSCLE (Multiple Sequence Comparison by Log-Expectation) [26]. The alignment was manually refined, and gaps were removed using BioEdit. The finished alignment was used to construct the phylogenetic tree using TREE-PUZZLE, a program to reconstruct phylogenetic trees from molecular sequence data by maximum likelihood method [27]. Additional trees were drawn using Parsimony (Paup 4.0) or Bayesian methods [28], [29].

### Methods to determine the mechanisms of duplication of *Trichomonas* TMAC genes

As described above, phylogenetic trees were drawn using cyclase domains to determine the number of ancestors for the present set of TMAC genes. To determine whether duplication of segments of chromosomes contributed to the large number of copies of TMAC genes, we aligned whole scaffolds (average size is ∼70,000 bp) containing TMAC genes with each other [8]. In the rare instances where there was extensive overlap in flanking sequences, we discriminated sequences that contained open reading frames versus those that contained repetitive elements. We also looked among the flanking sequences (as much as 40 kb on the two sides) for repetitive families, mobile elements, and microsatellites, as defined in the NCBI annotation of the *Trichomonas* scaffolds [8]. We looked for examples of gene conversion using the set of 11 programs included in the Recombination Detection Program (RDP) [30]. We also used the program GeneConv to detect gene conversion [31]. Gene conversion events were called when the majority of the different programs identified the event.

### Methods to identify pseudogenes among TMAC genes and other *Trichomonas* gene families

To identify TMAC pseudogenes, we took advantage of the absence of introns in any of the TMAC genes and the strict conservation of N-terminal TMHs and C-terminal cyclase domain in the predicted transmembrane cyclases [8], [11]. Most of the TMAC pseudogenes were identified using the complete TMAC protein sequence (TVAG_350120) and TBLASTN to search the scaffolds or contigs of *Trichomonas* at the JCVI or NCBI. Pseudogenes contained in-frame stop codons (nonsense mutation) and/or frame shifts that we could confirm by examining multiple independent primary sequence reads. In addition, we amplified the DNA around numerous of these stop codons by PCR to confirm their presence in the genome project G3 strain and to assess their occurrence in the B7RC2 and S1 strains. We also mapped the location of the various stop codons and frame shifts to determine whether any of them were present in more than one TMAC gene. This result would suggest that a pseudogene was duplicated. TMAC genes that were incomplete because they were at the edge of a contig were not considered pseudogenes.

Additional pseudogenes were identified using the paralog and ortholog function at TrichDB [21]. Briefly, ∼175 predicted proteins of *Trichomonas*, many of which were given different names (e.g. adenylate cyclase, guanylate cyclase, conserved hypothetical protein, etc.), were identified as paralogs or orthologs of the complete TMAC (TVAG_350120). TMAC pseudogenes were strongly suggested when these paralogs were present in an array of short proteins that spanned the length of a complete TMAC gene. In this case, the in-frame stop codons and/or frame shifts could be inferred by the prediction of multiple short proteins rather than a single full-length protein. Because stop codons and frame shifts in these pseudogenes identified using the paralog data base were not checked versus single reads, these pseudogenes are listed as putative in File S1.

While TMAC pseudogenes were identified by inspection, pseudogenes in cyclic nucleotide phosphodiesterases and other proteins in Table 1 were identified using a custom BLASTX and FASTX program that uses a protein template to look for in-frame stop codons or frame shifts in genomic DNA. In each case, we confirmed the stop codon or frame shift by examining multiple independent primary sequence reads in the GSS database at NCBI.

**Table 1:** Presence of pseudogenes in representative families of duplicated genes of *Trichomonas*.

protein family	average length in aa	family size	assembly boundary	genes with stops or FS^a^	truncated genes^b^	percentage of pseudogenes^c^
Dynein heavy chain family protein	3937	22	1	0	1	5%
transmembrane adenylyl cyclases	1550	123	12	56	4	46%
cyclic nucleotide phosphodiesterase	1134	41	2	1	7	18%
Clan SB, family S8, subtilisin-like serine peptidase	868	31	6	2	2	16%
Adaptin N terminal region family protein	811	51	2	3	1	6%
ABC transporter family protein	614	64	7	11	8	32%
Dolichol-phosphate-mannose-protein mannosyltransferase	479	31	0	1	1	6%
major facilitator superfamily protein	403	48	1	9	1	21%
Clan CA, family C1, cathepsin L-like cysteine peptidase	286	44	2	1	6	17%
small Rab GTPase	203	184	3	3	3	3%
small GTP-binding protein	193	39	0	1	2	5%
ADP-ribosylation factor	181	24	0	2	0	8%

a FS: frame shift.

b truncated genes: those whose length is between 30% to 70% of the length of a complete gene.

c pseudogenes: those containing stops and/or frame shifts and/or truncations that cannot be explained by assembly issues.

### Growth and cloning of *Trichomonas*

The S1 strain of *Trichomonas vaginalis*, was received from Dr. B. N. Singh (SUNY Health Science Center, Syracuse, New York), while the genome project G3 strain and B7RC2 strain were from Patricia Johnson (UCLA). *Trichomonas* was grown at 37°C and sub-cultured every 24 hr in TYI-S-33 medium containing 10% adult bovine serum [32]. *Trichomonas* was diluted in medium to 10^2–3^ cells/ml and cloned on plates containing 0.6% agarose [33]. *Trichomonas* was grown for seven days under anaerobic conditions. Individual clones were picked and sub-cultured in liquid medium in 48-well tissue culture plates, and RNA was isolated as described in the next section.

### RNA isolation and qRT-PCR

Total *Trichomonas* RNA isolated using the RNAqueous-4PCR kit (Ambion) was treated with DNAse1 for 1 hr at 37°C. First strand cDNA synthesis was performed with RETROscript (Ambion), using oligo dT primers for 1 hr at 42°C on ∼1 g RNA. PCR of *Trichomonas* cDNAs was performed using SYBR Green Master Mix with Rox from Roche Applied Science. Reverse transcriptase and template were separately omitted from negative controls, while primers to an actin gene (TVAG_094140) were positive controls for RT-PCR. For primer sequences used in the RT-PCRs, please see Data S2.

### Recombinant expression of *Trichomonas* cyclases and measurement of cyclase activities

Genomic DNA was isolated from one confluent flask (∼2×10^6^) of *Trichomonas*, using the Wizard Genomic DNA purification kit (Promega). PCR primers were designed to isolate representative DNAs encoding cyclase domains of two *Trichomonas* TMACs (TVAG_013980 and TVAG_456550). These PCR products were cloned into the pGEX-6p vector (Amersham Biosciences) [34]. *Escherichia coli* BL21 cells transformed with pGEX-6p were grown in LB medium and induced with 1 mM IPTG for 3 hrs at 30°C. Recombinant glutathione-S-transferase (GST)-cyclase fusion-proteins were purified with glutathione-agarose beads and released with 10 mM glutathione.

Cyclase activities of GST-fusion enzymes were measured as described in [35], and the colorimetric readout was measured according to manufacturer's instructions contained in adenosine 3′,5′-cyclic monophosphate (cAMP) and guanosine 3′,5′-cyclic monophosphate (cGMP) direct immunoassay kits (Biovision Research products, CA). Each reaction contained 4 µg of GST-fusion protein and 2 mM ATP and 0.2 mM GTP when assaying for cAMP, or 2 mM GTP and 0.2 mM ATP when assaying for cGMP. A positive control was the manufacturer's enzyme, while a negative control was GST alone. Reactions were diluted and measured versus cAMP or cGMP standards according to manufacturer's instructions.

### Bioinformatic identification of *Trichomonas* genes encoding cAMP phosphodiesterases

Putative *Trichomonas* cyclic nucleotide phosphodiesterases were searched using *Homo sapiens* sequences [22], [36]. Many of these putative phosphodiesterases were already predicted at TrichDB [21]. Cyclic nucleotide phosphodiesterase trees were made based on the amino acid sequences of conserved domain using the same methods as for the cyclase trees.

## Results

### Identification of a large family of *Trichomonas* genes encoding transmembrane cyclases

Using cyclase domains from TMACs of *Dictyostelium discoideum*, *Homo sapiens*, and *Trypanosoma brucei*, we identified ∼123 putative transmembrane cyclases in the predicted proteins of *Trichomonas* (Data S1) [8], [12]–[14], [21]. The few *Trichomonas* cyclases that lack a set of TMHs appear to be truncated versions of the same gene family or to be present at the edge of a contig (and so are incomplete because of assembly issues) [8]. Each complete transmembrane cyclase is ∼1450 to ∼1700 amino acids long and contains a series of six or eight TMHs at the N-terminus (Fig. S1) [23]. These TMHs are followed by an ∼300-aa domain that is relatively well conserved and predicted to be cytosolic. Four or six TMHs separate two extracellular domains. Finally, a microbial type 3 cyclase domain is present at the C-terminus in the cytosol [12].

Very similar cyclase domains are also present at the 3′ ends of ESTs of *Tritrichomonas foetus* and *Paratrichomonas hominis* (data not shown). Because the 5′ ends of these ESTs were not sequenced, it is not possible to confirm that the entire TMAC genes are conserved in these other trichomonads. With the exception of the cyclase domain, there is no similarity between the predicted transmembrane cyclases of *Trichomonas* and the transmembrane cyclases of metazoans and protists unrelated to *Trichomonas* (e.g. *Trypanosoma* or *Plasmodium*) [12]–[15]. We conclude that all the duplications of the transmembrane cyclase genes likely occurred in trichomonads rather than in a common ancestor to all eukaryotes.

### The large TMAC gene family results from the recent duplication of a small set of ancestral genes in trichomonads

**Figure 1:**
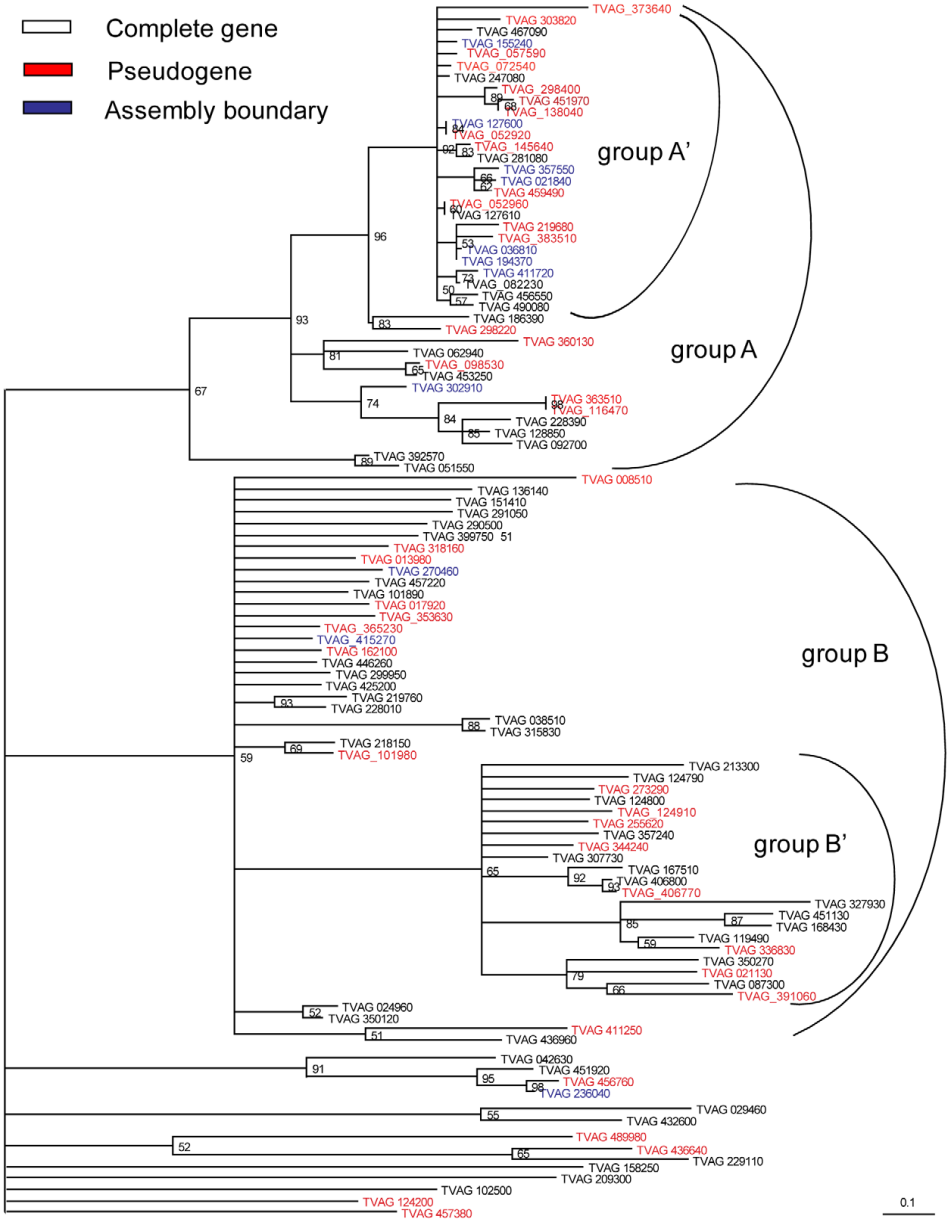
Transmembrane cyclase genes of *Trichomonas* form two groups. A phylogenetic tree constructed by maximum likelihood methods shows the cyclase domains of representative *Trichomonas* TMACs form two groups (A and B). A subgroup of A (A′) is most recently duplicated (shows very short branch lengths that are proportional to differences between sequences). Pseudogenes (marked in red) are present in both groups A and B. Incomplete genes due to assembly issues are marked in grey. Numbers at nodes indicate boot strap support for 100 iterations, while nodes with less than 50% support are collapsed. Similar results were obtained when trees were drawn using parsimony or Bayesian methods.

**Figure 2:**
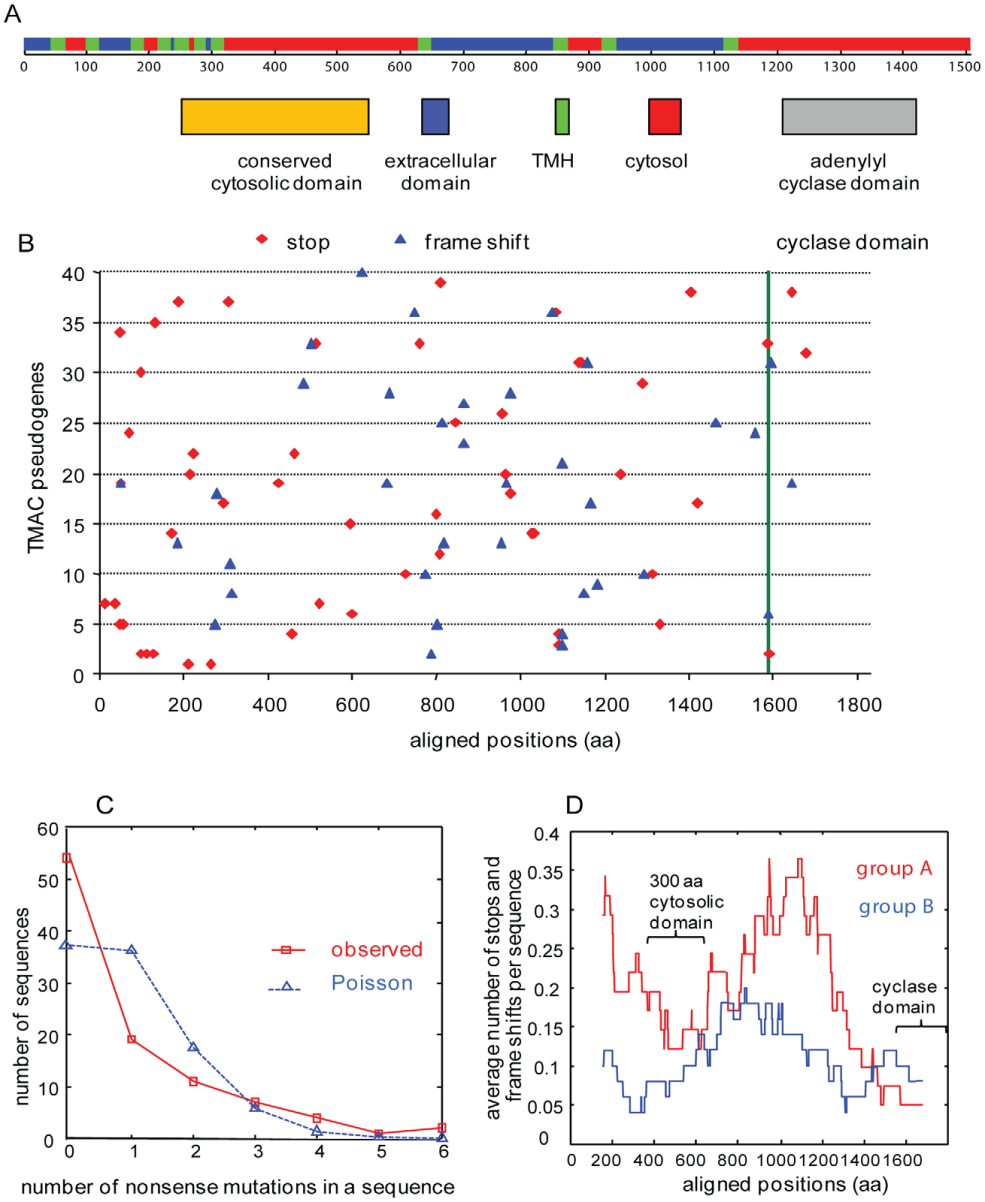
The distribution of stop codons (nonsense mutations) in TMAC genes differs from chance in multiple ways. A. Each transmembrane adenylyl cyclase (TMAC) has a similar topology predicted by Phobius. There is a series of N-terminal TMHs (green), followed by a conserved cytosolic domain (tan) and a C-terminal cyclase domain (grey) [23]. B. With one exception that may reflect an assembly error, none of the stops and frame shifts is in the same place in 40 aligned TMAC pseudogenes. This result suggests that the stop codons and frame shifts occurred after gene duplication. Note that the alignment technique, which introduces gaps, makes the TMACs appear longer than 1600 amino acids. Green line marks the beginning of the conserved cyclase domain. C. The actual distribution of stop codons among TMAC pseudogenes differs from a Poisson distribution. This result suggests there is selection against the first stop codon (disabling the protein coding capacity of the gene) but not against subsequent stop codons. D. Average number of stops and frame shifts are calculated using a window size of 300 aa in the aligned sequences of group A and B. Stop codons are more abundant in TMAC from the more recently duplicated group A than from group B. Stop codons are also more frequent in less conserved parts of the TMAC genes.

We used phylogenetic methods to show that representative TMAC genes fall into two major groups of roughly equal size (Fig. 1). *Trichomonas* TMAC genes in A′ sub-group are more recently duplicated (i.e. show shorter branch lengths) than other members of group A and those of group B. While we used maximum likelihood methods to make the tree shown in Fig. 1, similar trees were produced using parsimony and Bayesian treeing methods [28], [29]. For numerous reasons, we think group A and group B TMACs are similar. The topology of groups A and B TMACs each matches that shown in Fig. 2A and Fig. S1, and groups A and B TMACs have similar percentages of pseudogenes and similar patterns of expression by RT-PCR (see below). In addition, recombinant cyclase domains from each group both have adenylyl cyclase activity (see below).

For comparison, we used the same phylogenetic methods to align 41 predicted cyclic nucleotide phosphodiesterases of *Trichomonas*, which are cytosolic enzymes that likely hydrolyze cAMP produced by TMACs (Fig. S2) [8], [21], [36]. Many of the putative cyclic nucleotide phosphodiesterase genes of *Trichomonas* appear to be the result of recent duplication of a single ancestral gene (group A in Fig. S2).

### Evidence for gene conversion in *Trichomonas* TMAC genes

We wished to determine, if possible, the mechanism(s) for duplication of the TMAC genes. For the most part, there is only a single TMAC gene on a contig. Multiple TMAC genes are present on the same contig in just 12 of 90 instances, and the TMAC genes are tandemly repeated in just four cases. Other *Trichomonas* genes are not repeated in these contigs, so they do not resemble the subtelomeric regions of *Plasmodium* chromosomes, where more than one gene family is repeated [37].

**Figure 3:**
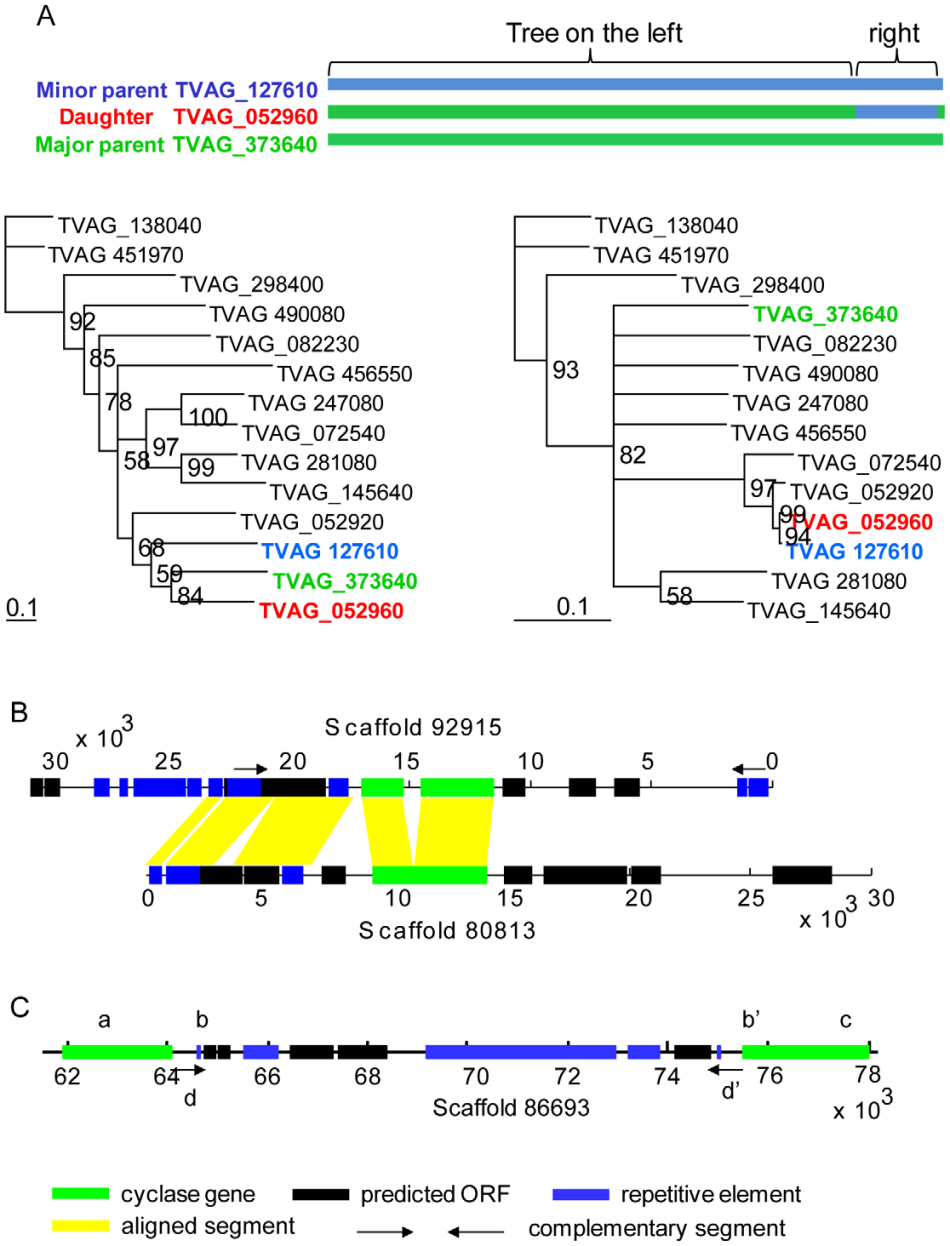
Rare examples of gene conversion of a *Trichomonas* TMAC gene, duplication of TMAC flanking sequences, and a repetitive element interrupting a TMAC gene. In A, the so-called daughter sequence (TVAG_052960 marked in red) is composed of two parts. The major portion of the daughter (green) derives from the so-called major parent (TVAG_373640), while the minor portion of the daughter (blue) derives from the so-called minor parent (TVAG_127610). In the tree on the left (representing the major portion), the daughter is more similar to the major parent than the minor parent. Conversely, the tree on the right (representing the minor portion), the daughter is more similar to the minor parent than the major parent. In B, predicted transmembrane cyclase genes (green) on two different scaffolds have similar flanking sequences on one side (marked with yellow lines). These flanking sequences including multiple ORFs (black) as well as repetitive elements (blue). Note there is a gap in the cyclase gene in scaffold 92915. This figure shows that a small segment of the chromosome that contains a transmembrane cyclase has been duplicated. In C, a single transmembrane cyclase (green) has been interrupted by a segment of DNA that contains predicted ORFs (black), repetitive elements (blue), and an inverted repeat (d and d′ at its ends).

There is strong evidence for a single gene conversion or a crossover event, in which both parent genes can be identified (Fig. 3A) [30], [31], [38]. In addition, there is indirect evidence for gene conversion, wherein the conserved cyclase domains of numerous TMAC pseudogenes have many fewer stop codons than non-conserved domains (Fig. 2 and see next section).

In about a dozen occasions, two TMAC genes each have the same flanking sequences that contain multiple open reading frames and short segments of repetitive DNA (Fig. 3B). In the vast majority of cases, however, only the coding sequences of the TMAC genes are duplicated. There are no particular microsatellites, repetitive DNAs, or mobile elements closely associated with the duplicated TMAC genes (Fig. S3) [8]. We identified a single occasion where a TMAC gene is interrupted by the insertion of a mobile element (Fig. 3C). The duplication of *Trichomonas* cAMP phosphodiesterase genes also appears to be independent of flanking sequences or repetitive elements (data not shown).

### A surprising number of *Trichomonas* TMAC genes contain stop codons and/or frame shifts and so appear to be pseudogenes

A high percentage of *Trichomonas* TMAC genes (∼46%) are pseudogenes, as they contain stop codons and/or frame shifts (the vast majority) or are truncated (the minority) (Figs. 1 and 2, Table 1, and Data S1). With one possible exception, these stop codons and frame shifts are unique, indicating that pseudogenes did not get duplicated. Conversely, the paucity of TMAC pseudogenes with many stop codons, frame shifts, and deletions suggests the possibility that older TMAC pseudogenes have been completely deleted from the *Trichomonas* genome. Similarly, the high percentage of synonymous versus non-synonymous mutations in the TMAC pseudogenes is consistent with the presence of recent purifying selection on these genes before they became pseudogenes [39]. The difference between the Poisson distribution and the actual distribution of the stop codons in TMAC genes suggests there is selection against the first in-frame stop, when protein-coding would be disturbed for the first time (Fig. 2C). TMAC pseudogenes are frequent in both group A and group B.

Stop codons in both groups A and B are less frequent in regions of the TMAC genes that encode the conserved domain of unknown function and cyclase domain (Fig. 2D). A possible explanation is gene conversion, wherein a segment of a wild-type sequence replaces the corresponding segment of a homologous pseudogene sequence [30], [31].

While the transmembrane cyclases have the highest percentage of pseudogenes (46%), 32% of ABC family transporters appear to be pseudogenes (Table 1). Other gene families have 16 to 18% pseudogenes (cathepsin L-like cysteine peptidases, subtilisin-like serine proteases, and cyclic nucleotide phosphodiesterases), while numerous gene families have <8% pseudogenes (Table 1). We did not attempt to estimate the overall rate of pseudogenes in the 60,000 predicted protein-encoding genes of *Trichomonas* [8], because many of these genes derive from Mavericks (giant transposable elements) [10] and we were unable to make protein models for many of the genes encoding hypothetical proteins.

### Stop codons in the TMAC genes are polymorphic among lab isolates of *Trichomonas*

**Figure 4:**
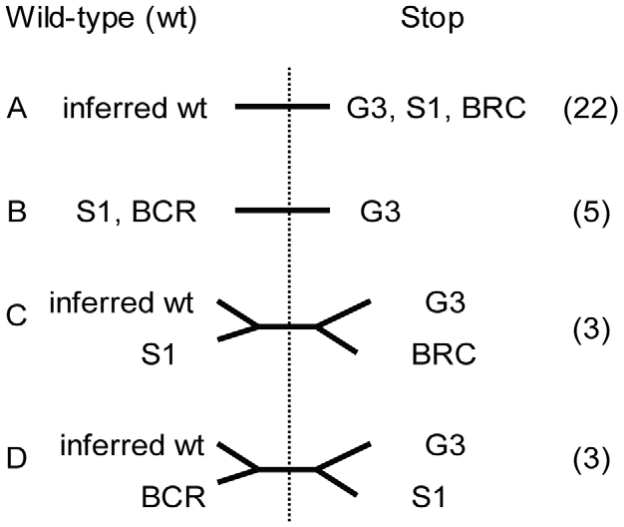
Phylogenetic trees or lines based upon polymorphisms at stop codons in TMAC genes of the *Trichomonas* genome project strain G3 demonstrate reassortment of markers. PCR products flanking 33 stop codons in TMAC genes were sequenced from S1 and B7RC2 strains of *Trichomonas* with the results graphed as follows. A. In 22 cases that are not informative concerning the history of the three strains, S1 and B7RC2 had the same stop codon as G3. This result indicates that the pseudogene was present in the common ancestor of all three strains, and we are unable to determine the wild-type sequence. In 11 cases that are historically informative (B to D) S1 and/or B7RC2 strains did not share the stop codon with G3. In these cases, we assume that the amino acid replacing the stop is the wild-type sequence. Interestingly, tree B suggests S1 and B7RC2 are more like each other than G3; tree C suggests B7RC2 is more like G3 than S1; while tree D suggests S1 is more like G3 than B7RC2. These findings that demonstrate reassortment of markers are inconsistent with clonal reproduction by *Trichomonas*, as has been suggested [40].

Many of the stop codons in the G3 TMAC genes (22 of 33 examined) are present in orthologous genes of two other *Trichomonas* strains (S1 and B7RC2) (Fig. 4A). This result suggests that these TMAC pseudogenes were present in the common ancestor of all three *Trichomonas* strains. In contrast, five stop codons are only present in the G3 strain, suggesting these stop codons have arisen more recently (Fig. 4B). Finally, there are six stop codons that are missing in either S1 or B7RC2, so the order of their divergence from the common ancestor is not resolved (Figs. 4C and 4D). Strict clonality, the presumed mode of reproduction in *Trichomonas* [40], cannot explain this pattern of stop codons in the three lineages.

### Multiple TMAC mRNAs (including those of pseudogenes) are expressed by cloned *Trichomonas*

**Figure 5:**
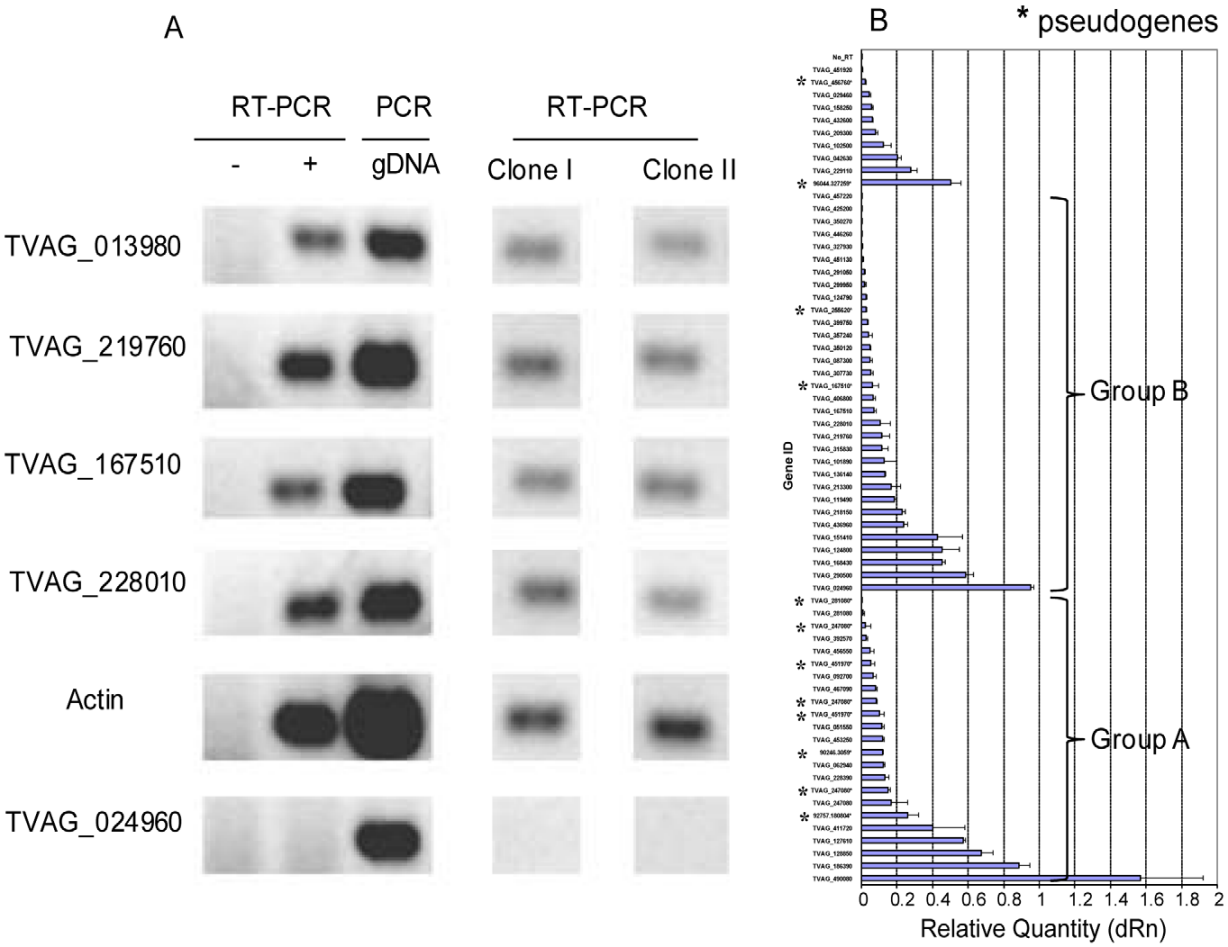
Messenger RNAs for numerous *Trichomonas* TMACs are expressed at the same time. A. RT-PCR shows that 4 of 5 TMACs are expressed by uncloned *Trichomonas* and by two clones of *Trichomonas* isolated on soft agar. B. qRT-PCR of uncloned *Trichomonas* shows that mRNAs vary in quantity more within particular group (A or B) of TMACs than they do between groups. The average abundance of mRNAs for TMACs pseudogenes is not statistically different than those for intact genes in either group A or group B (see Data S2 for gene and primer identifications).

Because there are so many different TMAC genes, we wondered whether multiple TMAC genes are expressed at the same time or whether a single TMAC gene is expressed at a time (variant expression). Variant expression has been described for surface antigens of *Giardia*, *Plasmodium*, and *Trypanosoma* [37], [41], [42]. In *Giardia* and *Plasmodium* variant expression occurs in part because there are different adherence functions to the surface proteins. Similarly, *Trichomonas* TMACs may have different functions in signal transduction. To begin to answer this question, we prepared mRNAs from two clones of *Trichomonas* that were isolated on soft agar [33]. RT-PCRs showed that 4 of 5 TMAC genes tested are expressed by each *Trichomonas* clone (Fig. 5A and Data S2). We used qRT-PCR to show that the abundance of TMAC mRNAs isolated from an uncloned population of *Trichomonas* varies widely (Fig. 5B). We found that there are greater differences between the expressions of mRNAs within a group (A or B) of TMACs than between groups A and B of TMACs. The expressions of 12 TMAC pseudogenes do not differ statistically from those of 53 intact TMAC genes. This result is consistent with the idea that nonsense mutations and frame shifts happened recently, so the promoters are still intact.

### Cyclase domains of representative *Trichomonas* transmembrane cyclases have adenylyl cyclase activity

Two cyclase domains from *Trichomonas* transmembrane cyclases, one arbitrarily chosen from group A (TVAG_456550) and one from group B (TVAG_013980), were expressed as glutathione-S-transferase (GST)-fusion enzymes in bacteria and incubated with ATP or GTP [34], [35]. Each recombinant *Trichomonas* cyclase showed adenylyl cyclase activity but no measurable guanylyl cyclase activity. For the group A cyclase, the K_m_ for ATP is 520±10 µM, and the specific activity is 6.1×10^−12^ mol/min/µg. For the group B cyclase, the K_m_ for ATP is 710±10 µM, and the specific activity is 8.5×10^−12^ mol/min/µg. We conclude that the *Trichomonas* transmembrane cyclases are adenylyl cyclases and have similar kinetics.

## Discussion

### Summary of the most important new findings

The very large genome of *Trichomonas* [8] may be partially explained by the presence of large, unstable families of genes such as those encoding TMACs that are undergoing massive gene duplication and concomitant development of pseudogenes (Figs. 1 and 2 and Data S1). Gene duplication and pseudogene formation both appear to be recent, as many TMAC genes are very similar to each other; numerous stop codons present in the genome project strain are not present in TMAC genes of other laboratory strains (Fig. 4); and mRNAs for many pseudogenes are still abundant (Fig. 5) [16]–[20].

Because we were unable to make good models for many of the unique *Trichomonas* proteins, we could not determine an overall rate of pseudogenes in *Trichomonas*. Based on the data in Table 1, though, it appears that the rate of *Trichomonas* pseudogenes is at least 5%. In GenBank there are 1354 *Trichomonas* genes annotated as pseudogenes (∼2% of the total 60,000 genes predicted) [8]. *Trichomonas* pseudogenes include 97 BspA genes, 42 kinases, 227 ankyrin repeat proteins, and 696 hypotheticals. However, only 5 of the 56 TMAC pseudogenes identified here are annotated as such in GenBank, suggesting the number of *Trichomonas* pseudogenes has been grossly underestimated. Regardless, the percentage of pseudogenes in *Trichomonas* is much greater than the percentages of pseudogenes (<0.1% in each) of protists with a similar microaerophilic life-style (*Giardia* and *Entamoeba*) [43]. Very high rates of pseudogenes, however, have been noted in proteins of *Trypanosoma cruzi* and *Trypanosoma brucei* that show variant expression [44], [45].

Stop-codons of TMAC pseudogenes are surprisingly polymorphic (Figs. 2 and 4) might be a useful target for studying the population biology of *Trichomonas*. The TMAC pseudogene sequences provide more precise information than methods that use restriction fragment length polymorphisms or pulse-field gel electrophoresis [46]–[48]. The TMAC pseudogene PCRs also demonstrate reassortment of polymorphic loci that cannot be explained by a strictly clonal reproduction of *Trichomonas* strains, as has been suggested [40]. While sexual reproduction (consistent with reassortment of genetic markers) has not been demonstrated in *Trichomonas*, the protist appears to have some of the conserved machinery for meiosis [8], [49]. Recent studies of *Giardia*, another microaerophilic protist, suggest there is reassortment of markers consistent with sex [50].

The *Trichomonas* cAMP-mediated signal transduction system predicted here differs in two fundamental ways from those of metazoans and *Dictyostelium* [12], [13], [51], [52]. First, the sequences the *Trichomonas* TMACs and cyclic nucleotide phosphodiesterases are unique. Second, *Trichomonas* TMACs and cyclic nucleotide phosphodiesterases are present in more copies than in metazoans, while predicted *Trichomonas* G protein-coupled receptors (GPCRs) are fewer than in metazoans (data not shown) [21], [53]. While the large number of TMACs in *Trichomonas* may be explained by their rapid duplication and concomitant conversion to pseudogenes, we cannot easily explain the relative paucity of GPCRs in *Trichomonas*. One possible explanation for the low rate of GPCRs is that the heterotrimeric G-proteins are activated independent of GPCRs, as has been noted in *Caenorhabditis elegans* [54]. Finally, there is genetic and biochemical evidence for heterotrimeric G-proteins that likely interact with Trichomonas TMACs [55], [56].

### Unresolved issues

The absence of synteny around most TMAC genes (Fig. 3) suggests gene duplication is not secondary to duplication of chromosomes or portions of chromosomes. The absence of repetitive elements around TMAC genes (Fig. S3) suggests these elements are not involved or are so unstable that they have been lost. Because only coding sequences of most TMAC genes are duplicated, it is possible that retrotransposition is involved. However, the absence of introns in duplicated TMAC genes cannot be used as an argument for retrotransposition, because the vast majority of *Trichomonas* genes lack introns [8], [11]. As many of the TMAC genes were recently duplicated, it was disappointing that we were unable to find a “smoking gun” that would provide the mechanism of duplication. In contrast, some of the 911 *Trichomonas* BspA genes are arranged in clusters with as many as 17 genes, consistent with several tandem duplication events [57].

The present studies cannot determine whether the TMAC pseudogenes are “junk” or have some function [16]. For example, by gene conversion (for which there is both direct and indirect evidence in *Trichomonas*) (Figs. 2 and 3), TMAC pseudogenes may be a source of alternative cyclase sequences for intact TMAC genes. Alternatively, TMAC pseudogene mRNAs (Fig. 5) may be involved in regulating expression of intact TMAC genes.

Most *Trichomonas* gene families do not have nearly the percentage of pseudogenes (46%) observed in TMAC genes (Table 1). Indeed some rather large gene families (e.g. Rab GTPases and small GTP-binding proteins) have very few pseudogenes. While these large families of *Trichomonas* genes certainly contribute to the enormous size of the genome, we do not know why there are so many copies of these genes.

The results of the RT-PCR (Fig. 5) suggest that multiple TMAC genes are expressed at the same time. We cannot rule out the possibility that some organisms under some conditions differentially express TMAC mRNAs, as these assays were performed with mRNA from single colonies containing a few thousand *Trichomonas* rather than mRNA of a single *Trichomonas*. We also tested the majority of TMAC mRNAs on uncloned protists, and trichomonads were all growing under similar culture conditions. However, variant expression, where each *Trichomonas* parasite expresses a single TMAC gene at a given time, seems unlikely.

Because there are so many TMAC genes, we assume that they play a role in pathogenesis [3], [7], [8], [58]. However, we do not know what signals are being transduced by TMACs. The whole genome sequence of *Trichomonas* also predicts a set of histidine kinases like those of bacteria and fungi [8], [59] but does not predict receptor-kinases that phosphorylate Ser, Thr, or Tyr (like those of metazoans and *Entamoeba)* [60], [61].

In summary, while the bioinformatic and experimental methods here have generated numerous novel findings concerning gene duplication and pseudogene development in *Trichomonas*, we are a long way from relating these findings to pathogenesis.

## Supporting Information

Data S1 Best estimate of the number of TMAC genes and pseudogenes.
(0.04 MB DOC)

Data S2 Primers used for RT-PCR of *Trichomonas* TMAC genes.
(0.03 MB XLS)

Figure S1 Sequence logo of aligned *Trichomonas* TMACs shows conserved domains. Seventy TMAC sequences were aligned, and the amino acid conservation (shown by the height of each position) was determined using WebLogo [29]. In particular, the C-terminal cyclase domain (grey) and conserved cytosolic domain of unknown function (tan) are well-conserved, indicating their importance for the function of the TMACs.
(1.20 MB TIF)

Figure S2 This figure, which complements Figure 1 in the main text, shows a phylogenetic tree constructed by maximum likelihood methods of cyclic nucleotide phosphodiesterases of *Trichomonas*. Pseudogenes are marked in red, while incomplete genes due to assembly issues are marked in grey. Branch lengths are proportional to differences between sequences, while numbers at nodes indicate boot strap support for 100 iterations. Nodes with less than 50% support are collapsed.
(0.37 MB TIF)

Figure S3 This figure, which complements Figure 2 in the main text, shows the relative paucity of microsattelites, repetitive elements, and mobile elements as defined in ref. [7] in sequences flanking *Trichomonas* transmembrane cyclase genes.
(0.23 MB TIF)

## References

[1] Johnston VJ, Mabey DC (2008). Global epidemiology and control of *Trichomonas vaginalis*.. Curr Opin Infect Dis.

[2] Glasier A, Gülmezoglu AM, Schmid GP, Moreno CG, Van Look PF (2006). Sexual and reproductive health: a matter of life and death.. Lancet.

[3] Schwebke JR, Burgess D (2004). Trichomoniasis.. Clin Microbiol Rev.

[4] Van Der Pol B, Kwok C, Pierre-Louis B, Rinaldi A, Salata RA (2008). *Trichomonas vaginalis* infection and human immunodeficiency virus acquisition in African women.. J Infect Dis.

[5] Hjort K, Goldberg AV, Tsaousis AD, Hirt RP, Embley TM (2010). Diversity and reductive evolution of mitochondria among microbial eukaryotes.. Philos Trans R Soc Lond B Biol Sci.

[6] Alsmark UC, Sicheritz-Ponten T, Foster PG, Hirt RP, Embley TM (2009). Horizontal gene transfer in eukaryotic parasites: a case study of *Entamoeba histolytica* and *Trichomonas vaginalis*.. Methods Mol Biol.

[7] Arroyo R, Gonzalez-Robles A, Martinez-Palomo A, Alderete JF (1993). Signalling of *Trichomonas vaginalis* for amoeboid transformation and adhesion synthesis follows cytoadherence.. Mol Microbiol.

[8] Carlton JM, Hirt RP, Silva JC, Delcher AL, Schatz M (2007). Draft genome sequence of the sexually transmitted pathogen *Trichomonas vaginalis*.. Science.

[9] Silva JC, Bastida F, Bidwell SL, Johnson PJ, Carlton JM (2005). A potentially functional mariner transposable element in the protist *Trichomonas vaginalis*.. Mol Biol Evol.

[10] Pritham EJ, Putliwala T, Feschotte C (2007). Mavericks, a novel class of giant transposable elements widespread in eukaryotes and related to DNA viruses.. Gene.

[11] Vanácová S, Yan W, Carlton JM, Johnson PJ (2005). Spliceosomal introns in the deep-branching eukaryote *Trichomonas vaginalis*.. Proc Natl Acad Sci U S A.

[12] Baker DA, Kelly JM (2004). Structure, function and evolution of microbial adenylyl and guanylyl cyclases.. Mol Microbiol.

[13] Kreibel PW, Parent CA (2004). Adenylyl cyclase expression and regulation during the differentiation of *Dictyostelium discoideum*.. IUBMB Life.

[14] Seebeck T, Schaub R, Johner A (2004). cAMP signalling in the kinetoplastid protozoa.. Curr Mol Med.

[15] Weber JH, Vishnyakov A, Hambach K, Schultz A, Schultz JE (2004). Adenylyl cyclases from *Plasmodium, Paramecium* and *Tetrahymena* are novel ion channel/enzyme fusion proteins.. Cell Signal.

[16] Balakirev ES, Ayala FJ (2003). Pseudogenes: are they “junk” or functional DNA?. Annu Rev Genet.

[17] Demuth JP, Hahn MW (2009). The life and death of gene families.. Bioessays.

[18] Zhou Q, Wang W (2008). On the origin and evolution of new genes–a genomic and experimental perspective.. J Genet Genomics.

[19] Zhang Z, Gerstein M (2004). Large-scale analysis of pseudogenes in the human genome.. Curr Opin Genet Dev.

[20] Ochman H, Davalos LM (2006). The nature and dynamics of bacterial genomes.. Science.

[21] Aurrecoechea C, Brestelli J, Brunk BP, Carlton JM, Dommer J (2009). GiardiaDB and TrichDB: integrated genomic resources for the eukaryotic protest pathogens *Giardia lamblia* and *Trichomonas vaginalis*.. Nucleic Acids Res.

[22] Altschul SF, Madden TL, Schaffer AA, Zhang J, Zhang Z (1997). Gapped BLAST and PSI-BLAST: a new generation of protein database search programs.. Nucleic Acids Res.

[23] Käll L, Krogh A, Sonnhammer ELL (2004). A combined transmembrane topology and signal peptide prediction method.. J Mol Biol.

[24] Fong JH, Marchler-Bauer A (2008). Protein subfamily assignment using the Conserved Domain Database.. BMC Res Notes.

[25] Crooks GE, Hon G, Chandonia JM, Brenner SE (2004). WebLogo: A sequence logo generator.. Genome Research.

[26] Edgar RC (2004). MUSCLE: multiple sequence alignment with high accuracy and high throughput.. Nucleic Acid Res.

[27] Schmidt HA, Strimmer K, Vingron M, von Haeseler A (2002). TREE-PUZZLE: maximum likelihood phylogenetic analysis using quartets and parallel computing.. Bioinformatics.

[28] Swofford DL (2003). PAUP*. Phylogenetic Analysis Using Parsimony (*and Other Methods). Version 4.

[29] Ronquist F, Huelsenbeck JP (2003). MRBAYES 3: Bayesian phylogenetic inference under mixed models.. Bioinformatics.

[30] Martin D, Rybicki E (2000). RDP: detection of recombination amongst aligned sequences.. Bioinformatics.

[31] Sawyer SA (1989). Statistical tests for detecting gene conversion.. Molecular Biology and Evolution.

[32] Clark CG, Diamond LS (2002). Methods for cultivation of luminal parasitic protists of clinical importance.. Clin Microbiol Rev.

[33] Philip A, Carter-Scott P, Rogers C (1987). An agar culture technique to quantitate *Trichomonas vaginalis* from women.. J Infect Dis.

[34] Smith DB, Johnson KS (1988). Single-step purification of polypeptides expressed in *Escherichia coli* as fusions with glutathione S-transferase.. Gene.

[35] Wiegn P, Dutton J, Lurie KG (1993). An enzymatic fluorometric assay for adenylate cyclase activity.. Analytical Biochem.

[36] Lu J, Bao Q, Wu J, Wang H, Li D (2008). CSCDB: the cAMP and cGMP signaling components database.. Genomics.

[37] Scherf A, Lopez-Rubio JJ, Riviere L (2008). Antigenic variation in *Plasmodium falciparum*.. Annu Rev Microbiol.

[38] Babushok DV, Ostertag EM, Kazazian HH (2007). Current topics in genome evolution: molecular mechanisms of new gene formation.. Cell Mol Life Sci.

[39] Cui J, Smith TF, Samuelson J (2007). Gene expansion in *Trichomonas vaginalis*: a case study on transmembrane cyclases.. Genome Inform.

[40] Tibayrenc M, Ayala FJ (2002). The clonal theory of parasitic protozoa: 12 years on.. Trends Parasitol.

[41] Prucca CG, Slavin I, Quiroga R, Elías EV, Rivero FD (2008). Antigenic variation in *Giardia lamblia* is regulated by RNA interference.. Nature.

[42] Figueiredo LM, Janzen CJ, Cross GA (2008). A histone methyltransferase modulates antigenic variation in African trypanosomes.. PLoS Biol.

[43] Pal D, Banerjee S, Cui J, Schwartz A, Ghosh SK (2009). *Giardia*, *Entamoeba*, and *Trichomonas* enzymes activate metronidazole (nitroreductases) and inactivate metronidazole (nitroimidazole reductases).. Antimicrob Agents Chemother.

[44] Arner E, Kindlund E, Nilsson D, Farzana F, Ferella M (2007). Database of *Trypanosoma cruzi* repeated genes: 20,000 additional gene variants.. BMC Genomics.

[45] Marcello L, Barry JD (2007). Analysis of the VSG gene silent archive in *Trypanosoma brucei* reveals that mosaic gene expression is prominent in antigenic variation and is favored by archive substructure.. Genome Res.

[46] Meade JC, de Mestral J, Stiles JK, Secor WE, Finley RW (2009). Genetic diversity of *Trichomonas vaginalis* clinical isolates determined by EcoRI restriction fragment length polymorphism of heat-shock protein 70 genes.. Am J Trop Med Hyg.

[47] Crucitti T, Abdellati S, Van Dyck E, Buvé A (2008). Molecular typing of the actin gene of *Trichomonas vaginalis* isolates by PCR-restriction fragment length polymorphism.. Clin Microbiol Infect.

[48] Upcroft JA, Delgadillo-Correa MG, Dunne RL, Sturm AW, Johnson PJ (2006). Genotyping *Trichomonas vaginalis*.. Int J Parasitol.

[49] Malik SB, Pightling AW, Stefaniak LM, Schurko AM, Logsdon JM (2007). An expanded inventory of conserved meiotic genes provides evidence for sex in *Trichomonas vaginalis*.. PLoS ONE.

[50] Cooper MA, Adam RD, Worobey M, Sterling CR (2007). Population genetics provides evidence for recombination in *Giardia*.. Curr Biol.

[51] Gilman AG (1987). G proteins: transducers of receptor-generated signals.. Annu Rev Biochem.

[52] Neves SR, Ram PT, Iyengar R (2002). G protein pathways.. Science.

[53] Davies MN, Gloriam DE, Secker A, Freitas AA, Mendao M (2007). Proteomic applications of automated GPCR classification.. Proteomics.

[54] Wilkie TM, Kinch L (2005). New roles for Galpha and RGS proteins: communication continues despite pulling sisters apart.. Curr Biol.

[55] Hirt RP, Lal K, Pinxteren J, Warwicker J, Healy B (2003). Biochemical and genetic evidence for a family of heterotrimeric G-proteins in *Trichomonas vaginalis*.. Mol Biochem Parasitol.

[56] Lal K, Noel CJ, Field MC, Goulding D, Hirt RP (2006). Dramatic reorganisation of *Trichomonas* endomembranes during amoebal transformation: a possible role for G-proteins.. Mol Biochem Parasitol.

[57] Noël CJ, Diaz N, Sicheritz-Ponten T, Safarikova L, Tachezy J (2010). *Trichomonas vaginalis* vast BspA-like gene family: evidence for functional diversity from structural organisation and transcriptomics.. BMC Genomics.

[58] Garcia AF, Alderete J (2007). Characterization of the *Trichomonas vaginalis* surface-associated AP65 and binding domain interacting with trichomonads and host cells.. BMC Microbiol.

[59] Wolanin PM, Thomason PA, Stock JB (2002). Histidine protein kinases: key signal transducers outside the animal kingdom.. Genome Biol.

[60] Blume-Jensen P, Hunter T (2001). Oncogenic kinase signalling.. Nature.

[61] Beck DL, Boettner DR, Dragulev B, Ready K, Nozaki T (2005). Identification and gene expression analysis of a large family of transmembrane kinases related to the Gal/GalNAc lectin in *Entamoeba histolytica*.. Eukaryot Cell.

