# Supplementary material for: Trichomonas Transmembrane Cyclases Result from Massive Gene Duplication and Concomitant Development of Pseudogenes
Source: PLoS Negl Trop Dis. 2010 Aug 3;4(8):e782. doi: 10.1371/journal.pntd.0000782 (PMC2914791; doi:10.1371/journal.pntd.0000782)
Supplement: Data S1 — Best estimate of the number of TMAC genes and pseudogenes. (0.04 MB DOC) [file pntd.0000782.s001.doc]

File S1: Best estimate of the number of TMAC genes (67) and TMAC pseudogenes (56). Annotations have been added to comments for each gene in TrichDB.

55 intact TMAC genes were identified using a model full-length TMAC ([TVAG_350120](http://trichdb.org/trichdb/addComment.do?stableId=TVAG_350120&commentTargetId=gene&externalDbName=T.+vaginalis+scaffolds&externalDbVersion=2007-01-11&organism=Trichomonas+vaginalis&locations=24628-29358&contig=DS113598&strand=-&flag=0&bulk=0)) and either BLASTP or TBLASTN of the predicted proteins or contigs, respectively, of *Trichomonas*. Designations are gene names in TrichDB:

TVAG_038510 TVAG_024960 TVAG_029460 TVAG_042630 TVAG_051550 TVAG_062940 TVAG_087300 TVAG_092700 TVAG_101890 TVAG_102500 TVAG_119490 TVAG_124790 TVAG_127610 TVAG_128850 TVAG_136140 TVAG_151410 TVAG_158250 TVAG_167510 TVAG_168430 TVAG_186390 TVAG_209300 TVAG_213300 TVAG_218150 TVAG_219760 TVAG_228010 TVAG_228390 TVAG_229110 TVAG_247080 TVAG_281080 TVAG_290500 TVAG_291050 TVAG_299950 TVAG_307730 TVAG_327930 TVAG_350120 TVAG_350270 TVAG_357240 TVAG_392570 TVAG_399750 TVAG_406800 TVAG_425200 TVAG_432600 TVAG_436960 TVAG_446260 TVAG_451130 TVAG_451920 TVAG_453250 TVAG_457220 TVAG_457380 TVAG_456550 TVAG_467090 TVAG_490080 [TVAG_010850](http://trichdb.org/trichdb/showRecord.do?name=GeneRecordClasses.GeneRecordClass&project_id=TrichDB&source_id=TVAG_010850) TVAG_013980 TVAG_235080

12 TMAC genes are partial due to assembly problems (e.g. at the edge of a contig or a gap in a contig):

TVAG_021840 TVAG_036810 TVAG_127600 TVAG_155240 TVAG_194370 TVAG_236040 TVAG_270460 TVAG_302910 TVAG_357550 TVAG_411720 TVAG_505880 TVAG_564290

43 TMAC pseudogenes were discovered using a model full-length TMAC ([TVAG_350120](http://trichdb.org/trichdb/addComment.do?stableId=TVAG_350120&commentTargetId=gene&externalDbName=T.+vaginalis+scaffolds&externalDbVersion=2007-01-11&organism=Trichomonas+vaginalis&locations=24628-29358&contig=DS113598&strand=-&flag=0&bulk=0)) and TBLASTN of the contigs in TrichDB. Stops, frame shifts, and/or truncations were all confirmed with primary sequence reads. Because numerous pseudogenes are identified as a series of genes in TrichDB, there are more than 43 “genes” listed below:

TVAG_021120 TVAG_021130 TVAG_298210 TVAG_298220 TVAG_298230 TVAG_456750 TVAG_456760 TVAG_008510 TVAG_008520 TVAG_013980 TVAG_017910 TVAG_017920 TVAG_124200 TVAG_124210 TVAG_124800 TVAG_124810 TVAG_162100 TVAG_162110 TVAG_162120 TVAG_194440 TVAG_219670 TVAG_219680 TVAG_255620 TVAG_255630 TVAG_273280 TVAG_273290 TVAG_303810 TVAG_303820 TVAG_315830 TVAG_318160 TVAG_318170 TVAG_318180 TVAG_336810 TVAG_336820 TVAG_336830 TVAG_344240 TVAG_344250 TVAG_353620 TVAG_353630 TVAG_353640 TVAG_360120 TVAG_360130 TVAG_360140 TVAG_360150 TVAG_363510 TVAG_411240 TVAG_411250 TVAG_436640 TVAG_451970 TVAG_451980 TVAG_459490 TVAG_459500 TVAG_489970 TVAG_489980 [TVAG_057580](http://trichdb.org/trichdb/showRecord.do?name=GeneRecordClasses.GeneRecordClass&project_id=TrichDB&source_id=TVAG_057580) [TVAG_057590](http://trichdb.org/trichdb/showRecord.do?name=GeneRecordClasses.GeneRecordClass&project_id=TrichDB&source_id=TVAG_057580) TVAG_298390 TVAG_298400 TVAG_298410 TVAG_124900 TVAG_124910 TVAG_124920 TVAG_373630 TVAG_373640 TVAG_373650 TVAG_373660 TVAG_373670 TVAG_391060 TVAG_391070 TVAG_391080 [TVAG_052900](http://trichdb.org/trichdb/showRecord.do?name=GeneRecordClasses.GeneRecordClass&project_id=TrichDB&source_id=TVAG_052900) [TVAG_052910](http://trichdb.org/trichdb/showRecord.do?name=GeneRecordClasses.GeneRecordClass&project_id=TrichDB&source_id=TVAG_052910) [TVAG_052920](http://trichdb.org/trichdb/showRecord.do?name=GeneRecordClasses.GeneRecordClass&project_id=TrichDB&source_id=TVAG_052920) [TVAG_052930](http://trichdb.org/trichdb/showRecord.do?name=GeneRecordClasses.GeneRecordClass&project_id=TrichDB&source_id=TVAG_052930) [TVAG_052940](http://trichdb.org/trichdb/showRecord.do?name=GeneRecordClasses.GeneRecordClass&project_id=TrichDB&source_id=TVAG_052940) [TVAG_052950](http://trichdb.org/trichdb/showRecord.do?name=GeneRecordClasses.GeneRecordClass&project_id=TrichDB&source_id=TVAG_052950) [TVAG_052960](http://trichdb.org/trichdb/showRecord.do?name=GeneRecordClasses.GeneRecordClass&project_id=TrichDB&source_id=TVAG_052960) TVAG_383490 TVAG_383500 TVAG_383510 TVAG_406770 TVAG_406780 TVAG_406790 TVAG_365230 TVAG_365240 TVAG_101980 TVAG_101990 TVAG_415270 TVAG_145630 TVAG_145640 TVAG_145650 TVAG_072530 TVAG_072540 TVAG_082220 TVAG_082230

TVAG_098510 TVAG_098520 TVAG_098530 TVAG_138020 TVAG_138030 TVAG_138040

13 putative TMAC pseudogenes were discovered using orthologs/paralogs file. Stops and/or frame shifts were not checked with primary reads. Again because numerous pseudogenes are identified as a series of genes in TrichDB, there are more than 13 “genes” listed below

[TVAG_034630](http://trichdb.org/trichdb/showRecord.do?name=GeneRecordClasses.GeneRecordClass&project_id=TrichDB&source_id=TVAG_034630) TVAG_095290 TVAG_095300 TVAG_102030 TVAG_102040 TVAG_116470 TVAG_116480 TVAG_147310 TVAG_147320 TVAG_158530 TVAG_158540 TVAG_158550 TVAG_162100 TVAG_162110 TVAG_162120 TVAG_406810 TVAG_358620 TVAG_358630 TVAG_420130 TVAG_425640 TVAG_431830 TVAG_431840 TVAG_431850 TVAG_489970 TVAG_489980 TVAG_284770 TVAG_284780
