# Supplementary figures and images for: Trichomonas Transmembrane Cyclases Result from Massive Gene Duplication and Concomitant Development of Pseudogenes
Source: PLoS Negl Trop Dis. 2010 Aug 3;4(8):e782. doi: 10.1371/journal.pntd.0000782 (PMC2914791; doi:10.1371/journal.pntd.0000782)

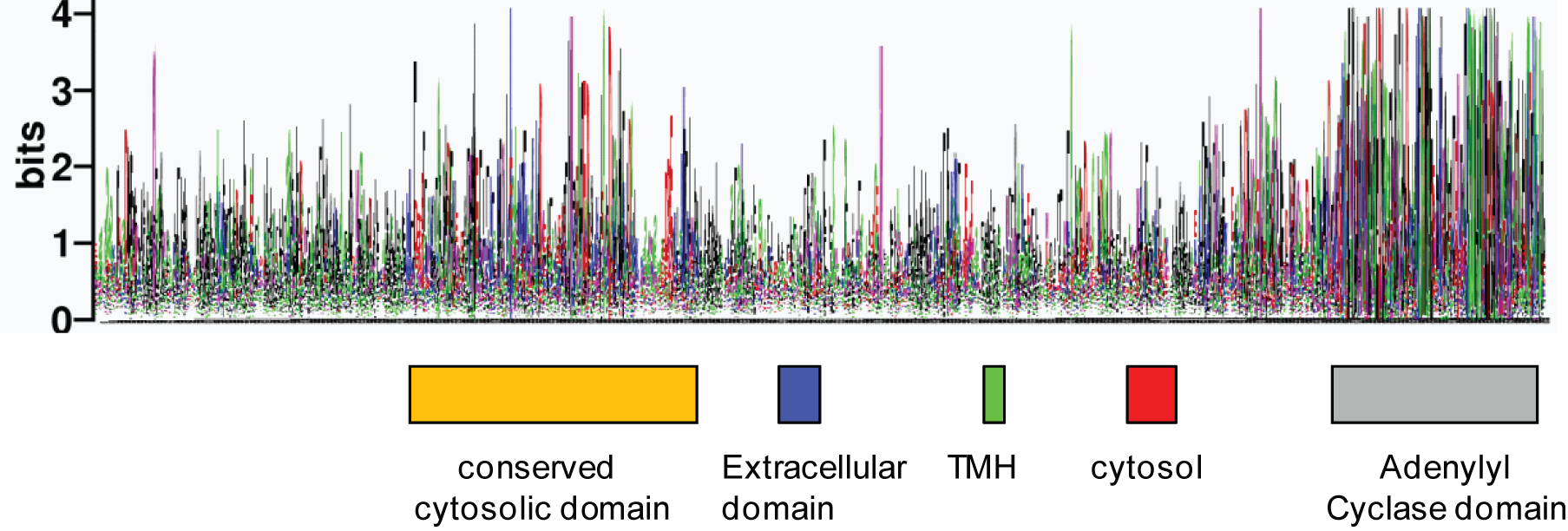

Supplement: Figure S1 — Sequence logo of aligned Trichomonas TMACs shows conserved domains. Seventy TMAC sequences were aligned, and the amino acid conservation (shown by the height of each position) was determined using WebLogo [29]. In particular, the C-terminal cyclase domain (grey) and conserved cytosolic domain of unknown function (tan) are well-conserved, indicating their importance for the function of the TMACs. (1.20 MB TIF) [file pntd.0000782.s003.tif]

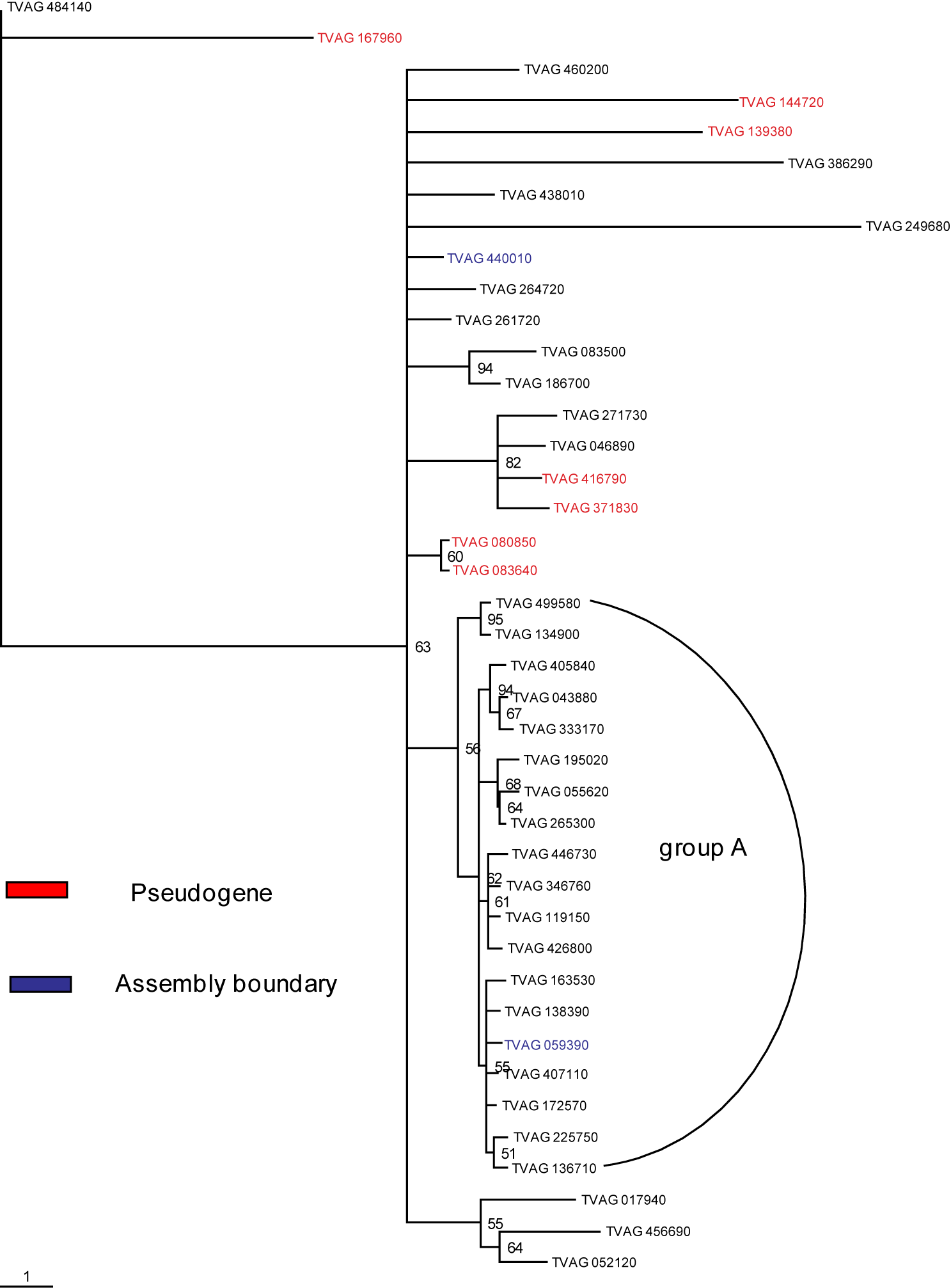

Supplement: Figure S2 — This figure, which complements Figure 1 in the main text, shows a phylogenetic tree constructed by maximum likelihood methods of cyclic nucleotide phosphodiesterases of Trichomonas. Pseudogenes are marked in red, while incomplete genes due to assembly issues are marked in grey. Branch lengths are proportional to differences between sequences, while numbers at nodes indicate boot strap support for 100 iterations. Nodes with less than 50% support are collapsed. (0.37 MB TIF) [file pntd.0000782.s004.tif]

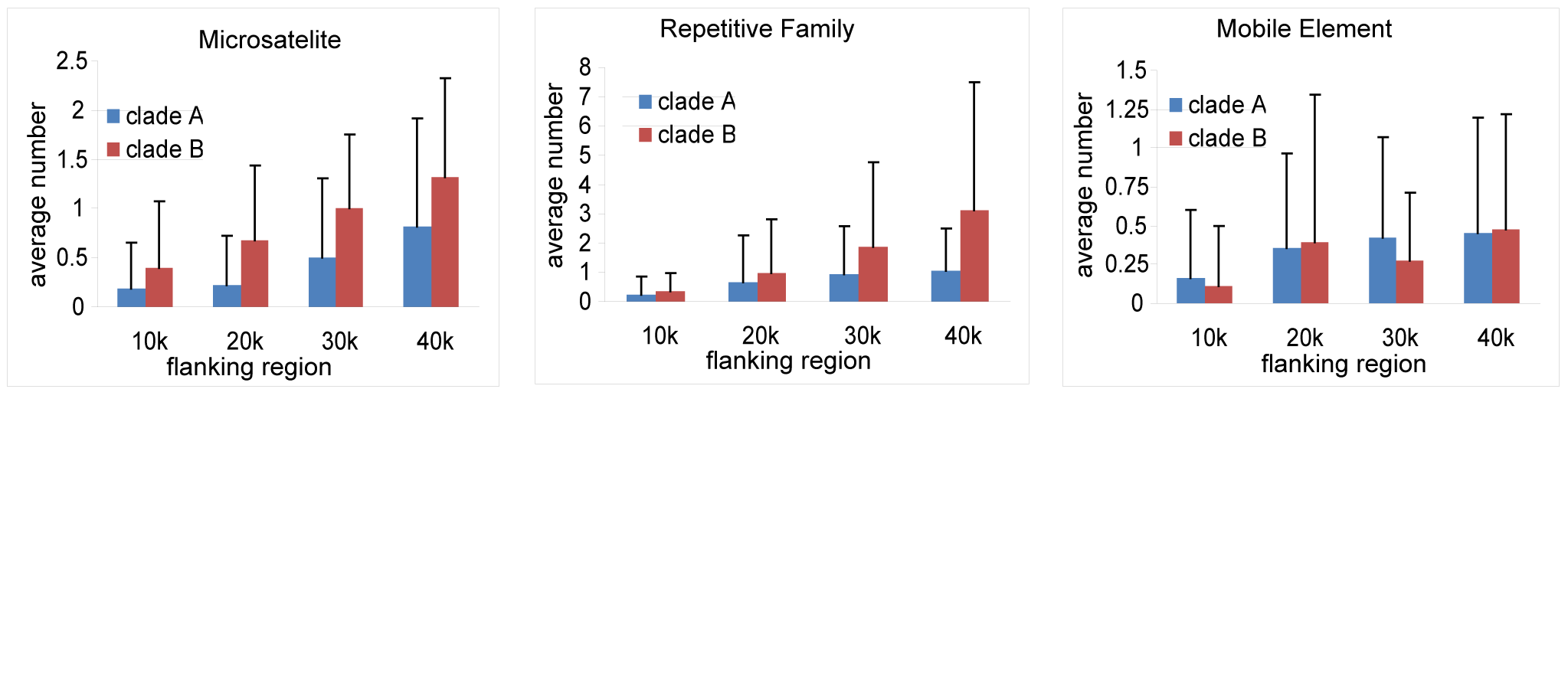

Supplement: Figure S3 — This figure, which complements Figure 2 in the main text, shows the relative paucity of microsattelites, repetitive elements, and mobile elements as defined in ref. [7] in sequences flanking Trichomonas transmembrane cyclase genes. (0.23 MB TIF) [file pntd.0000782.s005.tif]
